# Intravenous parecoxib sodium as an analgesic alternative to morphine in acute trauma pain in the emergency department

**DOI:** 10.1186/1865-1380-7-2

**Published:** 2014-01-03

**Authors:** Kamarul Aryffin Baharuddin, Nik Hisamuddin NA Rahman, Shaik Farid Abdull Wahab, Nurkhairulnizam A Halim, Rashidi Ahmad

**Affiliations:** 1 Department of Emergency Medicine, School of Medical Sciences, Universiti Sains Malaysia, Health Campus, 16150 Kelantan, Malaysia; 2 Department of Emergency Medicine, Hospital Tuanku Fauziah, 01000 Perlis, Malaysia; 3 Department of Emergency Medicine, University Malaya Medical Centre, 59100 Kuala Lumpur, Malaysia

## Abstract

**Background:**

Parecoxib sodium is the first parenteral COX-2 inhibitor used for pain management licensed for postoperative pain. However, no study has assessed the usage of parecoxib for acute traumatic pain in the emergency department (ED). The objective of this study was to investigate a potential alternative analgesic agent in the ED by determining the mean reduction of pain score between acute traumatic pain patients who were administered with intravenous (IV) parecoxib sodium versus IV morphine sulfate. The onset of perceptible analgesic effect and side effects were also evaluated.

**Methods:**

A randomized, double-blinded study comparing IV parecoxib 40 mg versus IV morphine at 0.10 mg/kg was conducted in adult patients presented with acute traumatic pain with numeric rating scale (NRS) of 6 or more within 6 hours of injury. Patients were randomized using a computer-generated randomization plan. Drug preparation and dispensing were performed by a pharmacist. Periodic assessment of blood pressure, pulse rate, oxygen saturation, and NRS were taken at 0, 5, 15, and 30 minute intervals after the administration of the study drug. The primary outcome was the reduction of NRS. Side effect and drug evaluation was conducted within 30 minutes of drug administration.

**Results:**

There was no statistically significant difference in the reduction of mean NRS between patients in the IV parecoxib group or IV morphine group (*P =* 0.095). The mean NRS for patients treated with IV morphine were 7.1 at 0 minutes, 4.5 at 5 minutes, 3.1 at 15 minutes, and 2.0 at 30 minutes. Whereas mean NRS for patients who received IV parecoxib were 7.8 at 0 minutes, 5.7 at 5 minutes, 4.7 at 15 minutes, and 3.9 at 30 minutes. The onset of perceptible analgesic effects could be seen as early as 5 minutes. Dizziness was experienced in 42.9% of patients who received IV morphine compared to none in the parecoxib group.

**Conclusions:**

There was non-significant trend toward superiority of IV morphine over IV parecoxib. Looking at its effectiveness and the lack of opioid-related side-effects, the usage of IV parecoxib sodium may be extended further to a variety of cases in the ED.

## Background

Parecoxib sodium is the first parenteral COX-2 inhibitor available for clinical use in pain management [1]. Based on data of clinical trials, its peak serum concentrations occur about 30 minutes after intravenous (IV) administration and 1 hour after intramuscular (IM) injection. Its first perceptible analgesic effect occurred within 7 to 13 minutes, with clinically meaningful analgesia demonstrated within 23 to 39 minutes and a peak effect within 2 hours following administration of single doses of 40 mg by IV or IM injection [2].

The efficacy of parecoxib sodium was established in multiple postoperative pain studies including gynecology and orthopedic surgery [3-7]. The analgesic efficacy of parecoxib sodium 20 and 40 mg, IV or IM, has been found to be similar to that of Ketorolac, 15 to 30 mg IV and 30 to 60 mg IM, and IV morphine 12 mg [3,4]. The advantages of this analgesic include its additive effect with morphine and its morphine-sparing effects as shown in multiple studies [5,6,8].

In the United Kingdom, IV parecoxib sodium is licensed for short-term treatment for postoperative pain [8]. A study regarding the use of parecoxib for postoperative pain showed that a single dose of parecoxib 20 mg or 40 mg provided effective analgesia for 50% to 60% of those treated compared to about 15% with placebo and was well tolerated [1]. Another multicenter study in Latin America looked at the use of parecoxib in acute renal colic and it showed parecoxib to be as effective as ketoprofen in the treatment of renal colic [9].

The appropriate usage of IV parecoxib sodium in the emergency department (ED) is not well established. To the best of our knowledge, there was no study regarding parecoxib sodium usage in the ED for acute traumatic pain. Therefore, the objective of this study was to investigate a potential alternative analgesic agent in the ED by determining the mean reduction of pain score between acute traumatic pain patients who were administered with IV parecoxib sodium versus IV morphine sulfate. Since time is essential in the ED, the onset of perceptible analgesic effect was determined and the side effects were evaluated.

## Methods

A randomized, double-blinded study comparing IV morphine sulfate at 0.10 mg/kg versus IV parecoxib sodium 40 mg was conducted in adult patients in the ED of Hospital Universiti Sains Malaysia (HUSM) from 1st August 2010 until 31st March 2011. HUSM is a tertiary centre located at the northeast of Peninsular Malaysia with 750 beds availabile for adult and pediatric patients. The annual census of the ED in 2009 was 56,662 cases, including all trauma and non-trauma cases. The annual census for orthopedic cases was 1,685 cases, which represents 3% of the total cases per annum [10]. Ethical approval was obtained from the Human Ethics Committee of Universiti Sains Malaysia and short-term research grant money from the university was used to buy the study drug (parecoxib sodium) as it was not a standard item in the ED.

Adult patients (aged 18 years and above) with significant acute traumatic pain who presented to the ED of HUSM within 6 hours of trauma were included. Significant acute traumatic pain was defined as pain score based on numeric rating scale (NRS) of 6 or more following trauma that involved bony fracture or soft tissue injury. Head injury cases with Glasgow Coma Scale ratings less than 15/15, intoxicated patients, pregnant patients, hemodynamically unstable patients, patients with a significant language barrier, or patients allergic to morphine, parecoxib sodium, or any non-steroidal anti-inflammatory drug or on any type of analgesia within the preceding 6 hours, were excluded.

All patients in the ED were triaged and the triager would alert the researchers once traumatic patients with a NRS of 6 or more had presented. Patients were brought to the treatment area by researchers and written informed consents were obtained. Focused history and physical examination were carried out by emergency residents on duty. Immobilization and splinting of the limbs and dressing of wounds were done according to the injuries. In the meantime, the pharmacist in charge was alerted regarding the patients’ body weight for drug randomization and preparation. All patients were randomized using a computer-generated randomization plan to receive IV parecoxib sodium 40 mg or IV morphine sulfate 0.1 mg/kg. To ensure researchers, emergency residents on duty, nurses, and patients were blinded to the treatment, the randomization was conducted by the pharmacist and he/she was responsible for preparation and dispensing of the study drug. The study drugs were administered by emergency residents once it was ready.

Periodic assessment of blood pressure, pulse rate, oxygen saturation, and NRS were taken at 0, 5, 15, and 30 minute intervals after the administration of the study drug. The assessment was conducted by the researchers who were kept blinded about the treatment. After 30 minutes of observation, if the NRS was 6 or more, a rescue drug (IV morphine sulfate 0.1 mg/kg) was administered to the patient by the emergency resident on duty. Side effects of the study drug were evaluated within 30 minutes of drug administration by asking the patients about any unwanted symptoms. Radiological investigation of the affected limb was taken, if indicated after 30-minute period of observation was over. Patients were then referred to the respective teams at the end of the study according to their injuries.

The primary outcome in this study was the reduction of pain score based on the NRS. The satisfaction of the study drug was assessed by asking patients “In terms of your satisfaction, how would you rate this pain medication?” 30 minutes after IV administration, using the scale “poor”, “fair”, “good”, and “excellent”.

We hypothesized that there would not be a significant trend towards IV morphine being superior to IV parecoxib for pain reduction in acute trauma pain in the ED. Data entry and analysis were done using the Statistical Package for Social Science (SPSS) version 12.0 software. The statistical analysis included descriptive analysis such as mean, standard deviation, percentage, paired *t*-test, and repeated measure ANOVA.

The parecoxib sodium used in this study was manufactured by Pharmacia & Upjohn Kalamazoo, Michigan, USA; it was produced for Pfizer using the trade name of Dynastat. Each vial of Dynastat contains 40 mg of parecoxib sodium for reconstitution. After reconstitution, the final concentration of parecoxib is 20 mg/mL. IV morphine sulfate was manufactured by Duopharma (M) Sdn Bhd, Selangor, Malaysia. Each milliliter of injection contained 10 mg of morphine sulfate. The dosage for IV morphine was 0.1 mg/kg. Once the dosage was calculated based on body weight, the pharmacist would prepare the morphine solution and dilute it to 2 mL of volume; this made parecoxib and morphine appeared identical.

## Results

A total of 32 patients, including 26 males and 6 females, were enrolled in the study. The youngest patient was 19 years old whereas the oldest was 65 years old. Eighteen patients received IV parecoxib sodium as an analgesic whereas 14 patients received IV morphine. Patients with a bone fracture were equal in number for both groups. Soft tissue injuries were administered parecoxib and morphine in 10 cases and 6 cases, respectively. Mean NRS were 7.1 ± 1.3 for the morphine group and 7.8 ± 1.3 for the parecoxib group (Table 1).

**Table 1:** Socio-demographic and clinical characteristic among the two different treatment groups

	**Morphine (n = 14)**	**Parecoxib sodium (n = 18)**
	**Mean (SD)/n (%)**	**Mean (SD)/n (%)**
**Age**	34.5 (14.7)	36.9 (15.3)
**Gender**		
Male	11 (78.6)	15 (83.3)
Female	3 (21.4)	3 (16.7)
**NRS**		
Mean (SD)	7.1 (1.3)	7.8 (1.3)
**Traumatic injury**		
Soft tissue injury	6 (42.9)	10 (55.6)
Bone fracture	8 (57.1)	8 (44.4)
**Side effect**		
Dizziness	6 (42.9)	0 (0.0)
Stomach upset	0 (0.0)	0 (0.0)
Allergic reaction	0 (0.0)	0 (0.0)
Nausea	0 (0.0)	0 (0.0)
No side effects	8 (57.1)	15 (83.3)
**Rescue drugs**		
Required	1 (7.1)	5 (27.8)
Not required	13 (92.9)	13 (72.2)
**Drug evaluation**		
Excellent	7 (50.0)	4 (22.2)
Good	7 (50.0)	11 (61.1)
Fair	0 (0.0)	2 (11.1)
Poor	0 (0.0)	1 (5.6)

**Figure 1:**
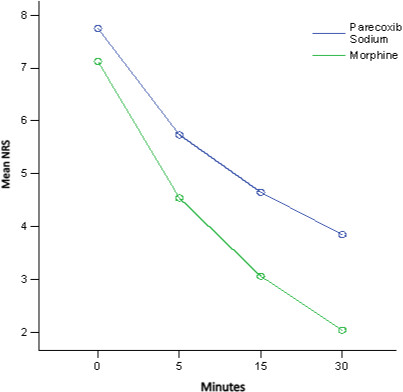
Estimated mean of NRS between patients treated with IV parecoxib sodium or IV morphine based on time.

There was no statistically significant difference in the reduction of mean NRS between patients that were treated with either IV parecoxib or morphine (*P* = 0.095). The mean NRS for patients that were treated with IV morphine were 7.1 at 0 minutes, 4.5 at 5 minutes, 3.1 at 15 minutes, and 2.0 at 30 minutes. Mean NRS for patients who received IV parecoxib were 7.8 at 0 minutes, 5.7 at 5 minutes, 4.7 at 15 minutes, and 3.9 at 30 minutes (Table 2). Figure 1 shows the estimated mean of NRS between patients treated with IV parecoxib or morphine within 0 to 30 minutes. The mean reduction of NRS for patients treated with IV parecoxib and IV morphine between 0–5 minutes, 0–15 minutes, 0–30 minutes, 5–15 minutes, 5–30 minutes, and 15–30 minutes were significant (Table 3).

**Table 2:** Comparison of mean NRS between patients treated with IV parecoxib sodium or IV morphine based on time

**Study drug**	**Time (minutes)**	**Mean (SD)**	**Estimated marginal mean (95** ***% *** **CI)**	P value*
**Parecoxib sodium**	0	7.8 (1.3)	7.09, 8.41	
	5	5.7 (2.2)	4.77, 6.71	
	15	4.7 (2.3)	3.66, 5.64	
	30	3.9 (2.3)	2.93, 4.77	
**Morphine**	0	7.1 (1.3)	6.38, 7.88	0.095
	5	4.5 (1.7)	3.44, 5.65	
	15	3.1 (1.6)	1.94, 4.19	
	30	2.0 (1.4)	0.99, 3.09	

*Repeated measure ANOVA.

**Table 3:** Comparison of mean reduction of NRS within patient treated with IV parecoxib sodium or IV morphine based on time

**Comparison**	**Parecoxib sodium**			**Morphine**		
**Time (minutes)**	**Mean (SD)**	**95% CI**	***P *** **value**	**Mean (SD)**	**95% CI**	***P *** **value**
**0–5**	2.0 (1.3)	1.34, 2.66	**<0.001**	2.5 (1.5)	1.70, 3.44	**<0.001**
**0–15**	3.1 (1.8)	2.24, 3.98	**<0.001**	4.1 (1.3)	3.34, 4.80	**<0.001**
**0–30**	3.9 (1.8)	3.05, 4.84	**<0.001**	5.1 (1.3)	4.31, 5.84	**<0.001**
**5–15**	1.1 (0.9)	0.66, 1.56	**<0.001**	1.5 (1.2)	0.83, 2.17	**<0.001**
**5–30**	1.9 (1.2)	1.37, 2.52	**<0.001**	2.5 (1.6)	1.57, 3.43	**<0.001**
**15–30**	0.8 (0.8)	0.44, 1.22	**<0.001**	1.0 (1.3)	0.25, 1.75	**0.013**

Dizziness was experienced by 42.9% of patients who received IV morphine compared to none in the parecoxib group; no other side effects were noted. Only 1 patient in the morphine group required rescue medication compared to 5 patients (27.8%) in the parecoxib group. All patients in the morphine group evaluated morphine as an excellent or good drug whereas only 15 patients in the parecoxib group evaluated parecoxib as an excellent or good drug (Table 1).

## Discussion

In the practice of emergency and acute care medicine, pain is the most frequent symptom in patients with a wide variety of injuries and illness. Indeed, numerous studies have proved the high prevalence of pain in the ED, ranging from 70% to 80% [11,12]. Thus, evaluation and management of acute pain is a fundamental element in the ED and acute care medicine. Good pain management is one of the major elements that correlate with patients’ satisfaction in the ED [13].

Even though the management of pain in the ED has improved with the usage of morphine as a gold standard for emergency acute pain relief [14,15], its side effects have limited its usage in polytrauma patients especially with concomitant head injury. It can cause nausea, vomiting, sedation, respiratory depression, delirium, and myoclonus [16]. The variability of prescribing opioid analgesics in painful conditions such as ankle fracture was present among emergency physicians from the American College of Emergency Physicians [17]. In fact, physicians’ preference toward non-steroidal anti-inflammatory drugs can be seen in some of the EDs [18].

The emergence of IV parecoxib sodium as a first parenteral selective COX-2 inhibitor has provided an alternative for the analgesic of choice for pain management especially in postoperative pain management. From our study, there was a non-significant trend toward superiority of IV morphine over IV parecoxib. The mean NRS at 30 minutes for the parecoxib group was 3.9 (SD ± 2.3) whereas that for the morphine group was 2.4 (SD ± 1.4). Even though there was no statistically significant difference between these two groups, patients who received IV morphine were able to achieve better pain control than those in the parecoxib group. All patients in the IV morphine group evaluated it as an excellent or good drug.

Both groups were also able to achieve significant pain reduction based on time within their groups (Table 2). Patients who received IV parecoxib showed a significant reduction from 0 to 5, 15, and 30 minutes. Meanwhile, patients who received IV morphine also showed a significant reduction for most of the time, except between 15–30 minutes. This is understood as IV morphine was able to reduce the magnitude of pain score faster than parecoxib (Figure 1). It also showed better reduction of NRS in the morphine group compared to the parecoxib group within the study period, even though the graph patterns were almost parallel. Figure 1 also shows that the perceptible analgesic effect occurred as fast as 5 minutes in the parecoxib group, which is comparable with a previous clinical study [2].

Based on the pain management research, both drugs had achieved minimal clinically significant difference (MCSD) of pain after treatment, even at 5 minutes after drug administration. Bijur et al. concluded that MCSD for NRS was 1.3 [19], whereas Kendrick and Strout found the NRS difference was 1.39 ± 1.05 [20]. Even though there was no statistically significant difference between IV parecoxib and IV morphine, and both drugs had achieved MCSD, one of the important factors to be determined was the magnitude of treatment effect that matters for patients in acute pain [21]. From Table 1 and Figure 1, IV morphine has shown to have better magnitude of pain reduction between these two drugs. In fact, there were 5 patients (27.8%) that required rescue medication in the parecoxib group.

However, almost 50% of patients who received IV morphine developed side effects of dizziness, whereas none in the parecoxib group did. This is comparable with a previous study that found no association with opioid related side-effects in the parecoxib group [3]. Nevertheless, one study noted that dizziness was the most frequently reported treatment-related side effect in comparing parecoxib and ketoprofen, with an incidence for dizziness in parecoxib vs. ketoprofen of 2.9% and 0.6%, respectively [9]. A possible explanation for the lack of side effects in the parecoxib group in this study is the short observation period, which was 30 minutes.

The present study was limited by the small sample size and it was likely underpowered to detect a significant difference. Furthermore, its observation period of 30 minutes was probably too short to determine the efficacy and side effects of parecoxib as its peak effect occurs within 2 hours of IV administration.

## Conclusions

IV parecoxib has potential as an analgesic alternative to morphine in acute trauma pain in the ED. Looking at its effectiveness and lack of opioid-related side-effects, the usage of IV parecoxib sodium may be extended further to a variety of cases in the ED. Its morphine-sparing effects are also an advantage to minimize the dose of morphine should the patient require opioids during the continuation of care in the emergency setting.

## Abbreviations

ED: Emergency department; HUSM: Hospital Universiti Sains Malaysia; IM: Intramuscular; IV: Intravenous; MCSD: Minimal clinically significant difference; NRS: Numeric rating scale.

## Competing interests

The authors declare that they have no competing interests.

## Authors’ contributions

KAB, NAH and SFAW designed the study, collected the data and analyzed the data. KAB and NAH drafted the manuscript. RA revised the study design, interpreted the results of the data analysis and revised the manuscript. NHNAR revised the data analyzed and revised the manuscript. All authors read and approved the final manuscript.
